# Exploring the morphology and taphonomy of *Archaeoniscus brodiei*—a gregarious, Early Cretaceous isopod

**DOI:** 10.1007/s00114-025-01962-8

**Published:** 2025-02-10

**Authors:** Russell D. C. Bicknell, Adiël A. Klompmaker, Patrick M. Smith, Thomas A. Hegna

**Affiliations:** 1 Division of Paleontology (Invertebrates), American Museum of Natural History, New York City, NY 10027 USA; 2 Palaeoscience Research Centre, School of Environmental and Rural Science, University of New England, Armidale, NSW 2351 Australia; 3 Department of Museum Research and Collections & Alabama Museum of Natural History, University of Alabama, Tuscaloosa, Alabama 35487 USA; 4 Palaeontology Department, Australian Museum Research Institute, Sydney, NSW Australia; 5 Department of Biological Sciences, Macquarie University, Sydney, NSW Australia; 6 Department of Geology and Environmental Sciences, State University of New York at Fredonia, New York, NY 14063 USA

**Keywords:** Isopoda, Taphonomy, Anatomy, Crustacea, Cretaceous, Gregarious behaviour

## Abstract

Marine isopod fossils represent a small component of the crustacean fossil record, contrasting the exceptional modern diversity of marine representatives of Isopoda. Examination of previously documented isopod species therefore presents an opportunity to derive additional paleobiological and taphonomic insight of these rare fossils. Here we consider two clusters of *Archaeoniscus brodiei* from the Lower Cretaceous (middle to upper Berriasian) Intermarine Member of the Durlston Formation, England. The individuals within the clusters are mostly complete, of similar size ranges, and are preserved on two different bedding planes. After examining these individuals, we illustrate, for the first time, appendages and eyes of *A. brodiei*. The appendage morphology supports the interpretation of *A. brodiei* as an isopod adapted to a benthic lifestyle. We propose that isopods from the Durlston Formation follow similar taphonomic pathways to arthropods preserved within plattenkalk-like deposits, resulting in enrichment in calcium carbonate and phosphate. Finally, the clusters reflect gregarious activities that were preserved during hypoxic events brought on by concurrent decay of algal blooms.

**Supplementary Information:**

The online version contains supplementary material available at 10.1007/s00114-025-01962-8.

## Introduction

Isopods are malacostracan crustaceans known from marine, freshwater, and terrestrial environments, both today and in the fossil record (Wieder and Feldmann 1992; Kensley 1998; Wilson 2008; Williams and Boyko 2012; Sfenthourakis and Taiti 2015; Becking et al. 2017). Modern isopods consists of over 10,000 species, demonstrating an exceptional diversity (Schotte et al. 2008; Wilson 2008; Poore and Bruce 2012; Hadfield and Smit 2020). Comparatively, the fossil record of isopods is limited, despite the group persisting since at least the Carboniferous (Schram 1970; Wieder and Feldmann 1992; Hyžný et al. 2013; Etter 2014; Robin et al. 2021). This may reflect the small (< 1 cm) size for some taxa (e.g., Fraaije et al. 2019). Despite this restricted record, continued examination of fossil isopods has revealed important aspects of their evolution (e.g., Schram and Abele 1982; Wilson and Edgecombe 2003; Nagler et al. 2017; Haug et al. 2021;), ontogeny (Schädel et al. 2021b; Van der Wal et al. 2021), palaeoecology (e.g., Wilson et al. 2011; Klompmaker and Boxshall 2015; Nagler et al. 2016; Stinnesbeck et al. 2022; Schädel et al. 2023), systematics (e.g., Wieder and Feldmann 1992; Wilson and Keable 2001; Etter 2014; Vonk et al. 2015), as well as terrestrialization (Broly et al. 2013) for extinct species.

The re-examination of well-known extinct isopods presents opportunities to further our understanding of the palaeobiology and preservational conditions of fossil forms. To expand the insight into extinct species, we examine individuals of *Archaeoniscus brodiei* Milne-Edwards, 1843, from the Purbeck Limestone Group, England. We explore the morphology and paleoecology of *A. brodiei*, and consider the taphonomic process that resulted in the isopod-rich beds from the so-called “Isopod Limestone” (Brodie 1845; Ross and Vannier 2002).

## Geological context

*Archaeoniscus brodiei* specimens have previously been collected from the Durlston and Lulworth formations of the Purbeck Limestone Group, England (Brodie 1845; Clements 1993; Ross and Vannier 2002). As these formations span the Jurassic–Cretaceous boundary, the slab studied herein may be from either time period. Importantly, metadata associated with the specimen report the Vale of Wardour section as the provenance. This information, combined with the presence of two isopod clusters that are similar to those in Ross and Vannier (2002), indicate the specimen was collected from the Isopod Limestone—beds within the Intermarine Member of the Durlston Formation (Ross and Vannier 2002). The slab is therefore likely to be Early Cretaceous (middle to late Berriasian) in age (Clements 1993; Ross and Vannier 2002; Schnyder et al. 2006; Joyce et al. 2022). Stratigraphically, the member is located above the Cinder Bed and below the Scallop Member, both of which represent marine incursions (Schnyder et al. 2006; Joyce et al. 2022).

The matrix surrounding the specimens consist predominately of well-bedded, ungraded, microcrystalline micritic limestone, with sparse fine quartz grains and clay particles. Given that the Intermarine Member is generally interpreted to represent a very shallow water deposit based on lithological and faunal characteristics (West 1979; Batten 2002), the fine-grained nature of these sediments suggests the fossils were deposited in relatively low energy conditions; potentially either a still-water marginal lagoon or freshwater carbonate lake (Feldmann et al. 1998; Buscalioni and Fregenal-Martínez 2006). The lack of grading (e.g. fining upwards) through the matrix associated with these isopods suggests they were not entombed rapidly in a turbiditic deposit. More likely, they died and were buried during a period when decay was inhibited, e.g. due to fluctuations in salinity and/or oxygen. The latter is more likely given previous work on the unit which has observed very high total organic carbon values, a high hydrogen index value, concentration of amorphous organic matter particles, and the presence of *Botryococcus*-type and *Celyphus rallus* algal body fossils (Riboulleau et al. 2007; Schnyder et al. 2009; Coram and Radley 2021). All these factors indicate the unit preserves the remains of partially decayed “algal blooms” (i.e. enhanced algal–bacterial biomass production) and fluctuating redox conditions in the water column.

## Methods

The examined slab is housed within the American Museum of Natural History, New York City, New York (prefix AMNH-FI) invertebrate paleontology collection and was assigned the specimen number AMNH-FI 15488. The examined specimens were assigned additional AMNH-FI numbers (AMNH-FI 142808—142813). The slab was imaged using an Olympus E-M1MarkIII camera with a 12–45 mm lens after the slab was coated with ammonium chloride. Images were made by stacking ten photographs using the OM Capture software. Specimen lengths were measured using ImageJ (Schneider et al. 2012). Close up images of specimens (AMNH-FI 142808—142812) were made with Cannon EOS Mark II 6D with a MP-E 65 mm lens set up as part of a Macropod stackshot system. Close up images were composed of multiple images taken at different depths of field and digitally stacked using Zerene Stacker to create one, hyperfocused image.

To explore the elemental composition and possible preservational mode of individuals within AMNH-FI 15488, the entire slab was examined using Scanning Electron Microscopy (SEM) Energy Dispersive X-Ray Spectroscopy (EDS). Individuals (AMNH-FI 142808, 142809, 142813) on the slab were analysed, uncoated, under low vacuum (30 kPa), nitrogen conditions at a voltage of 20 kV and an amperage of 3 nA, using a Tescan Vega S5124 at the SUNY Fredonia to produce a series of elemental maps and back scatter electron (BSE) images. Elemental map images were gathered using an Oxford Ultimax 65 mm detector at a resolution of 4096 K pixels, processed and exported on AZtec software (version 6.1/Oxford).

## Results

Isopods on AMNH-FI 15488 are ovate, dorsally compressed exoskeletons, with a subquadrate cephalic regions (Ross and Vannier 2002). These characteristics are comparable to those in specimens that have been referred to as *Archaeoniscus brodiei* in the published literature (Brodie 1839, 1845; Fisher 1856; Ross and Vannier 2002; Coram and Jepson 2012). Given these systematic similarities, and the previous identification of *A. brodiei* from the Purbeck Limestone Group, we assign the examined individuals to this taxon.

**Fig. 1:**
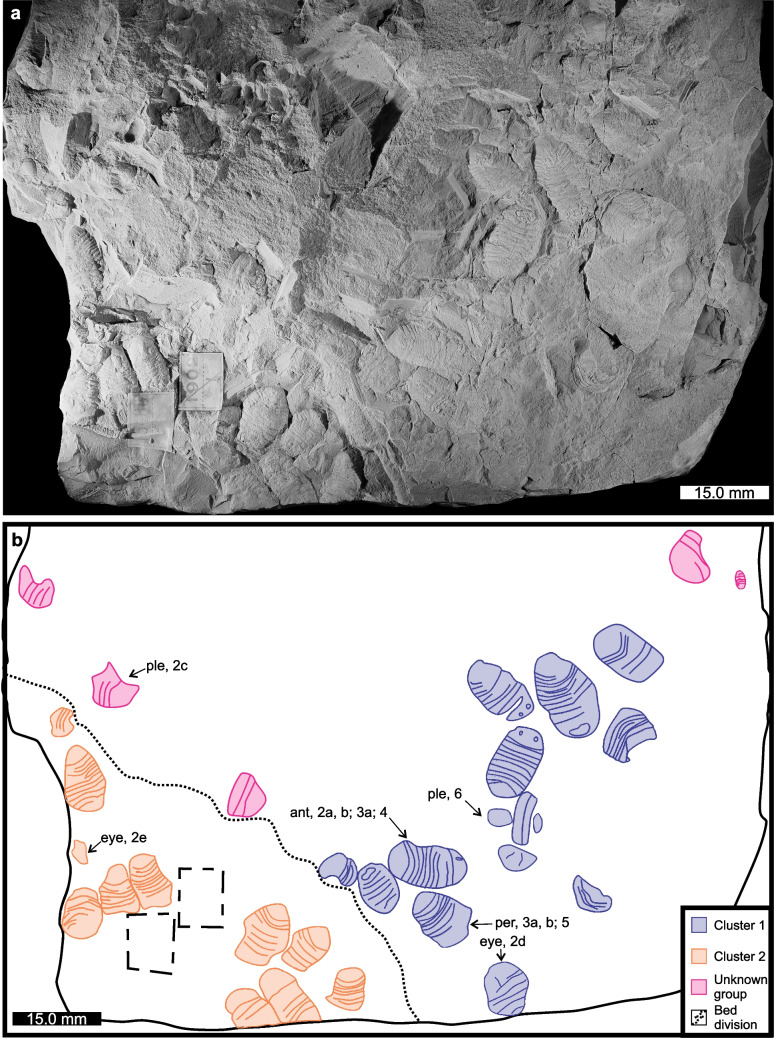
Depiction of the examined slab showing two clusters of *Archaeoniscus brodiei*. **a** AMNH-FI 15488. **b** Line drawing of the two clusters of isopods showing the bedding plane division. The two clusters are colour coded (blue and orange). Additional fragmented specimens are also shown (pink). Dotted line highlights the differentiation between the upper (blue) and lower (orange) beds and associated clusters. Dashed line indicates label locations. (**a**) coated in ammonium chloride and image converted to greyscale. Abbreviations: ant: antennulae and antennae; eye: lateral compound eye; per: pereiopods; ple: pleon with mottled cuticle morphology. Numbers and letters denote close ups in Figs. 2–6

The isopods are preserved as parts on two bedding planes in two clusters (Fig. 1). A total of seven complete individuals, at least 16 mostly complete individuals, and 4 fragments are observed in the two clusters. Additionally, four fragmentary exoskeletal sections and one juvenile individual are observed on other bedding planes. Complete individuals in the two clusters range in length between 9.4–15.1 mm and incomplete individuals range from 3.5–8.0 mm.

**Fig. 2:**
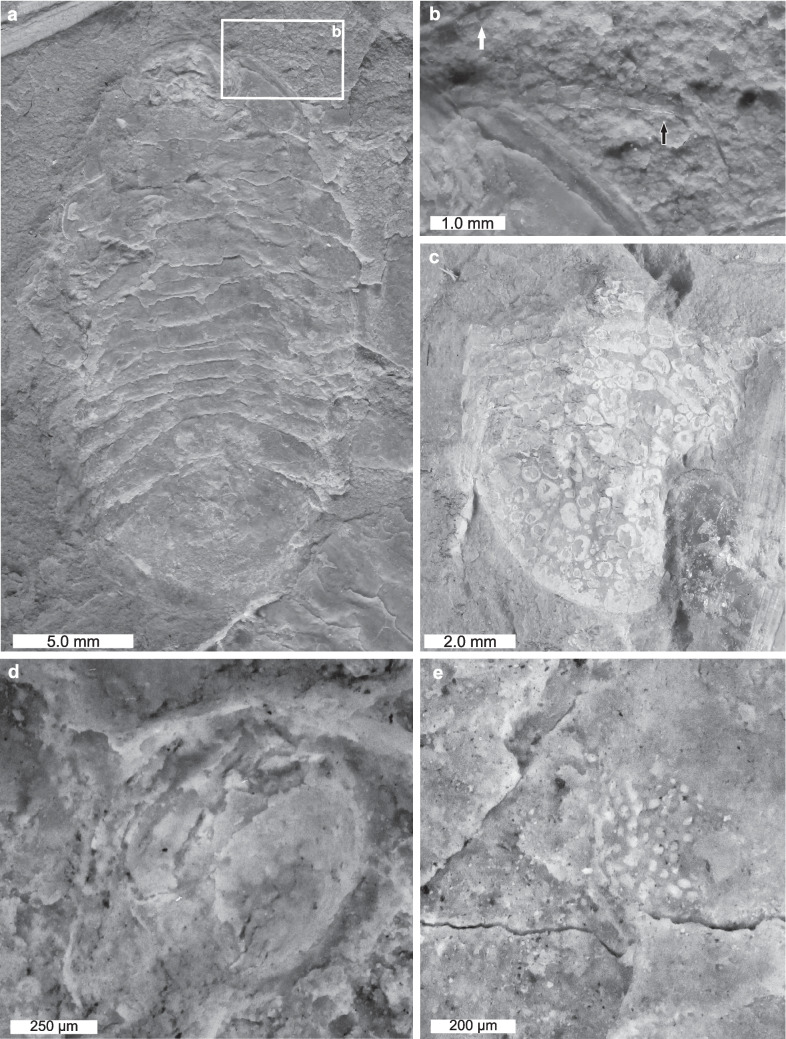
Evidence for detailed *Archaeoniscus brodiei* anatomy. **a**, **b** Individual showing antennulae and antennae. AMNH-FI 142808. **a** Complete individual. **b** Close up of antennulae (white arrow) and antennae (black arrow). **c** Pleon section showing mottled morphology. AMNH-FI 142810. **d**, **e** Lateral compound eye. **d** Lateral compound eye with faint ommatidia. AMNH-FI 142811. **e** Lateral compound eye with pronounced ommatidia. AMNH-FI 142812

**Fig. 3:**
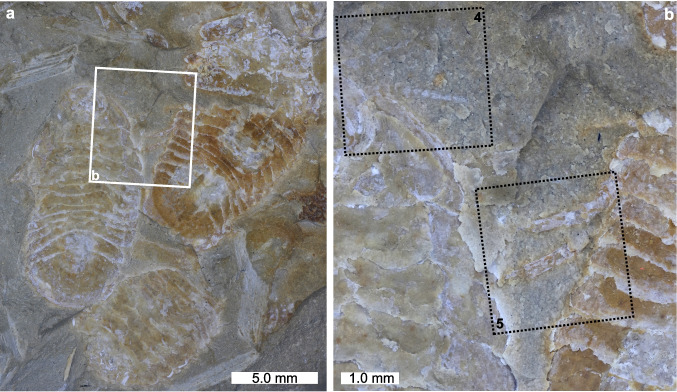
Evidence for *Archaeoniscus brodiei* pereiopods, antennulae and antennae. **a**, **b** Three specimens showing detailed appendicular morphologies. **a** Complete image. **b** Close up of box in (**a**) showing pereiopods, antennulae and antennae. AMNH-FI 142808, AMNH-FI 142809. Black boxes numbered 4 and 5 indicate areas mapped in Figs. 4 and 5, respectively

**Fig. 4:**
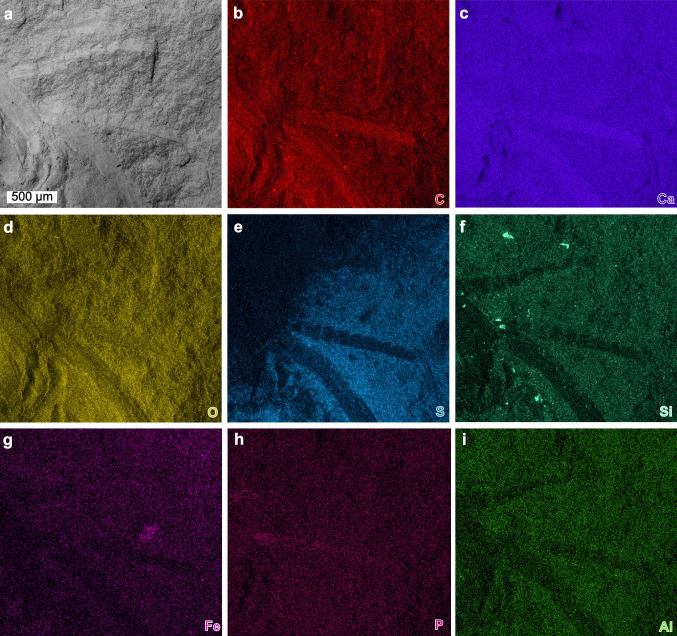
SEM backscatter image and EDS elemental maps of antennulae and antennae; area indicated in Fig. 3B. AMNH-FI 142808. **a** Backscatter image of antennulae and antennae. **b**–**i** Elemental maps of carbon, calcium, oxygen, sulphur, silica, iron, phosphorus and aluminium, respectively

**Fig. 5:**
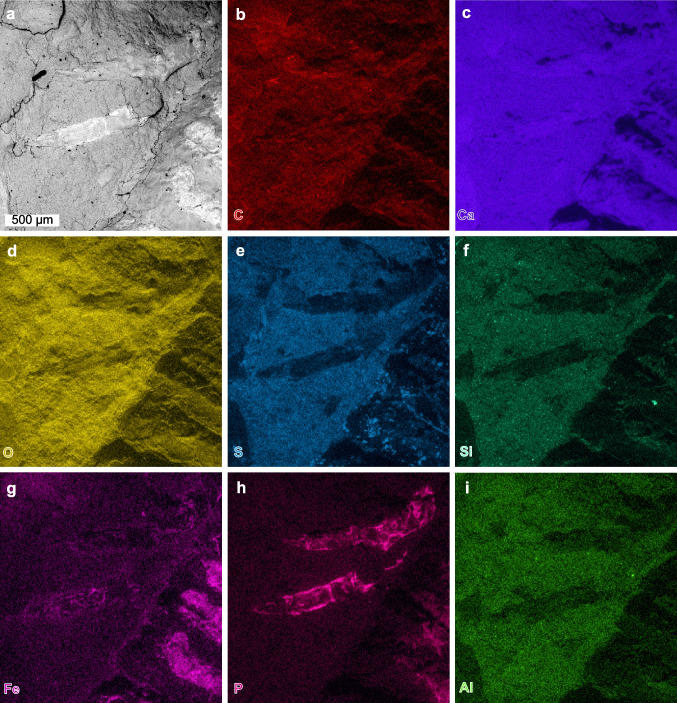
SEM backscatter image and EDS elemental maps of pereiopods; area indicated in Fig. 3B. AMNH-FI 142809. **a** Backscatter image of pereopods. **b**–**i** Elemental maps of carbon, calcium, oxygen, sulphur, silica, iron, phosphorus and aluminium, respectively

**Fig. 6:**
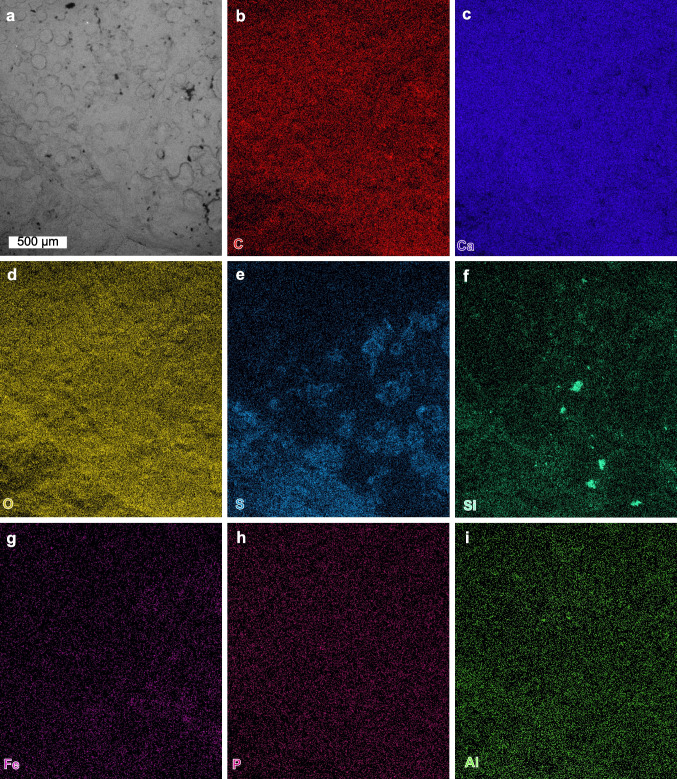
SEM backscatter image and EDS elemental maps of a mottled pleon. AMNH-FI 142813. **a** Backscatter image of antennae. **b**–**i** Elemental maps of carbon, calcium, oxygen, sulphur, silica, iron, phosphorus and aluminium, respectively

Examining individuals in detail uncovers novel anatomical data. Two individuals showed antennulae and antennae preserved outside the exoskeletal dorsum (Figs. 2a, b, 3a, b, 4) and one individual shows evidence of pereopods (Figs. 3a, b, 5). The antennula has only one article visible and a flagellum composed of a minimum of seven articles. The antennae has only one article visible, and a flagellum composed of a minimum of eight articles. The pereiopods (probably corresponding to pereiopods VI and VII) are stenopodous with a triangular terminal dactylus. Two examples of poorly preserved lateral compound eyes are identified with ommatidia preserved (Figs. 2d, e). Finally, two isolated pleon sections showing a mottled morphology are identified, one with a uropod preserved (Figs. 2c, 6). The mottled texture seems to be a result of taphonomic demineralization. The uropod has spine-like uropodal rami.

Elemental maps highlight distinct preservational aspects for the dorsal exoskeleton, appendages, and the matrix (Figs. 4–6). Antennae show enrichment in calcium and carbon, with limited enrichment in phosphorus (Fig. 4b, c, h). Walking legs show a marked enrichment in calcium and phosphorus, with a limited enrichment in carbon and iron (Fig. 5b, c, g, h). Pleon tergites show enrichment in sulphur and phosphorus, with limited enrichment in calcium and carbon (Fig. 6e, f). The pleon section with a mottled morphology shows enrichment in oxygen, sulphur, and silica. The matrix around the fossil shows pervasive enrichment of oxygen, sulphur, silica and aluminium (Figs. 4–6).

## Discussion

### Comments on morphology

*Archaeoniscus* is known from four currently accepted species. The generic position of *A. texanus* Wieder and Feldmann, 1992, was questioned by Park et al. (2012). However, Jones et al. (2014) tacitly accepted both *A. texanus* and the long-synonymized *A. edwardsi* Westwood, 1854, as valid species without comment or justification. We accept the better justified species list of Park et al. (2012). The most widespread of this is the type species, *A. brodiei*. First recognized from the Lower Cretaceous Purbeck strata in England in the 1840s (Milne-Edwards 1843; Brodie 1845; McCoy 1849), it was subsequently identified from three Upper Jurassic units: the Serpulit/Münder marls of west-central Germany (Haack 1918; Paproth 1956), the Abu Ballas Formation in Egypt (Klitzsch et al. 1979; Park et al. 2012), and the Cazels Formation in France (Margérard 2000; Gaillard et al. 2005). The only detailed attempt at systematically describing *A. brodiei* from material from the Purbeck Limestone was by Milne-Edwards (1843), which was then largely repeated by McCoy (1849). Milne-Edwards (1843) mentioned in his description that he did not observe *A. brodiei* appendages, but Reverand Brodie had. These appendages have never been described. As a result, the description that has been used to identify *A. brodiei* elsewhere in Europe is schematic. Indeed, *A. brodiei* has never had a holotype specimen assigned. The lot of specimens depicted in the first illustrations of *A. brodiei* (Brodie 1845) that would be a likely source for a lectotype are housed in the Natural History Museum in London (Ross and Vannier 2002). A redescription and restudy based on material from the Purbeck Limestone including type material is needed to better assess whether the older occurrence from the ?Tithonian of Germany (Paproth 1956) are truly conspecific, as well as the designation of a lectotype.

### Taphonomy and preservation

The examined isopods show a preservation that is typical of original crustacean and insect cuticle (Wang et al. 2009; Oliveira et al. 2015; Osés et al. 2016; Bezerra et al. 2018, 2020; Iniesto et al. 2019). Enrichment in calcium and phosphorus in regions of cuticle likely represent calcium phosphate replacement (apatite: Ca_5_(PO_4_,CO_3_)) (Martill 1988; Briggs and Wilby 1996; Wilby and Briggs 1997; Feldmann et al. 2012). Phosphate enrichment likely occurred soon after death, during early diagenesis, as bacterial mediated mineralisation is associated with fluctuating redox and/or during periods of prolonged oxygen depletion (Briggs et al. 1993; Schiffbauer et al. 2014; Muscente et al. 2015, 2019; Sinha et al. 2021). This is the likely situation within the Intermarine Member. Similar replacement is now well documented in numerous other arthropods preserved within similar plattenkalk-style deposits (Briggs et al. 1993, 2005; Wilby and Briggs 1997; Bicknell et al. 2019b, 2021; Muscente et al. 2019). Furthermore, the enrichment of aluminium, carbon, oxygen, and silica in the matrix aligns with an expected limestone elemental composition (Šiler et al. 2018), with the limited sulphur enrichment reflecting variation in a limestone matrix (Kramar et al. 2011).

With this pathway in mind, the enrichment of iron in the pereopods is somewhat anomalous, especially because iron enrichment is associated with depletion of phosphorus and sulphur. The replacement of elements by iron in fossils has previously been ascribed to pyrite oxidation (Osés et al. 2016; Gueriau et al. 2020) and pyrite framboids have been documented in Durlston Formation vertebrate fossils (Martill and Unwin 1997). Pyrite would be expected in oxygen depleted sediments of the Intermarine Member. As such, limited pyrite oxidation may have produced this pattern. However, remobilisation of iron through weathering is also a plausible explanation here (Alexander 1959), as iron is generally rather labile in the environment under surface conditions. Due to this uncertainty, we highlight both as viable options to explain the local enrichment of iron.

### Clustering

Clusters of multiple arthropods that are approximately the same size, species, preserved on the same bedding plane are called ‘body clusters’ and reflect mating, moulting, or other gregarious activities, such as clustering for protection (Speyer and Brett 1985; Karim and Westrop 2002; Paterson et al. 2007, 2008; Gutiérrez-Marco et al. 2009; Radwanski et al. 2009; Brett et al. 2012; Klompmaker and Fraaije 2012; Haug et al. 2013; Brett 2015; Bicknell et al. 2019a; Schwimmer and Montante 2019; Corrales-García et al. 2020; Hsieh and Plotnick 2020; Bicknell and Kimmig 2023). Such accumulation were likely preserved during mass mortality events such as either sudden burial and/or extreme changes in water chemistry (i.e. dissolved oxygen or salinity) contemporaneous with the behavioural aggregation of the organisms (Brett et al. 2006; 2012). The presence of two clusters on AMNH-FI 15488, which occur in separate, distinct bedding planes indicates that two gregarious events were preserved under similar depositional conditions. Furthermore, the preservation of mostly articulated *Archaeoniscus brodiei* individuals in approximately life orientation (not overturned or enrolled) suggests biological aggregations, rather than mechanical accumulation (El-Shahat and West 1983; Feldmann et al. 1998; Gaillard et al. 2005; Park et al. 2012). The lack of graded bedding documented here suggests that sudden burial and sediment smothering (e.g. during a storm event) is unlikely to have preserved these clusters. A more likely scenario is the isopod groupings were killed during mass mortality events following a sudden algae bloom causing stratification of the water column. This interpretation is strongly support by the geochemical evidence for hypoxia in the Intermarine Member (see comments under the geological context section). Similar isopod mortality events have also been observed and modelled in modern species populations resulting from decay of algal blooms (Martins et al. 1997; Ferreira et al. 2004). Further study of the redox-sensitive trace elements and isotopes in these isopod bearing beds could confirm this hypothesis.

At least 24 records of isopod biological aggregations have been considered (Tab. 1). Although explanations for this clustering behaviour is often limited, when presented it is thought to reflect scavenging associated with a larger organism (Polz 2004; Wilson et al. 2011; Robin et al. 2019) or looking for a final host in parastic forms (Schädel et al. 2021a). Past records of ‘Isopod Limestone’ clusters have no evidence of carcasses proximal to any documented associations (Brodie 1845; Ross and Vannier 2002) and this is confirmed herein. Additionally, as *Archaeoniscus* has mandibles morphologically comparable to modern sphaeromatid isopods (compare Park et al. 2012 to Bowman 1985), forms that are commonly herbivores or detrital feeders (Holdich and Jones 1973), we exclude scavenging as an explanation here. There is limited evidence for moulting in the isopods on the slab (Sahadevan et al. 2022). This excludes moulting as a primary explanation for the cluster (Speyer and Brett 1985; Daley and Drage 2016). We therefore propose that the clusters reflect sheltering (Broly et al. 2016) or possibly mating. This conclusion aligns with suggestions that articulated isopod fossils associated with fragments record a sudden hypoxia event after algal bloom decay with limited transport (Wieder and Feldmann 1992).

**Table 1:** Summary of previously documented fossil isopod clusters and possible explanations. Records presented from old to young. '–' indicates that no explanation was presented. '*' thought to be Tithonian to Berriasian in age in the original publications

Species	Stratigraphic unit and geography	Stage	Explanation	Citation
**Late Jurassic**				
*Archaeoniscus brodiei* Milne-Edwards, 1843	Serpulit/Münder Marls, Germany	? Tithonian	—	Paproth (1956)
*Archaeoniscus* sp.	Cazals Formation, France	Tithonian	—	Gaillard et al., (2005)
A member of Aegidae or *Tridentella* sp.	Solnhofen Limestone, Germany	Tithonian	Scavenging on arthropod carcass	Polz (2004)
**Early Cretaceous**				
*Archaeoniscus brodiei*	Durlston Formation, Purbeck Limestone Group, UK	Berriasian	Shelter or mating	Brodie (1839, 1845); Fisher (1856); Ross and Vannier (2002); Coram and Jepson (2012); this paper
*Cirolana enigma* Wieder and Feldmann 1992	Lakota Formation, South Dakota, USA	Berriasian–Valanginian	—	Wieder and Feldmann, (1992)
‘Isopod crustaceans’	North Germany	?Berriasian﻿–Aptian	?Current aligned, turbidite	Brett and Seilacher (1991)
‘*Archaeoniscus brodiei*’	Abu Ballas Formation, SW Egypt	Aptian*	—	Barthel and Boettcher (1978); Klitzsch et al., (1979)
*Brunnaega tomhurleyi* Wilson et al., 2011	Toolebuc Formation, Queensland, Australia	Albian	Scavenging on vertebrate carcass	Wilson et al., (2011)
*Archaeoniscus aranguthyorum* Feldmann et al., 1998	Tlayúa Formation, Puebla, Mexico	Albian	—	Feldmann et al. (1998); Vega et al., (2005, 2019)
**Late Cretaceous**				
*Cryptolacruma nidis* Schädel et al., 2021a	Burmese Amber, Kachin State, Myanmar	Cenomanian	?Presence of a host	Schädel et al. (2021a)
*Unusuropode castroi* Duarte and da Silva Santos, 1962	"Açu Arenite", Apodi Group, North Brazil	Turonian	—	Duarte and da Silva Santos (1962)
**Eocene**				
*Cirolana titanophila* Robin et al., 2019	Bolca Konservat-Lagerstätte, Mote Bolca, Italy	Ypresian	Scavenging on electric rays	Robin et al., (2019)
*Eosphaeroma margarum* (Desmarest, 1822)	Bembridge Insect Bed, Bembridge Marls, Isle of Wight, UK	Priabonian	—	Woodward (1879); Martini (1972); Gallego et al., (2019)
**Oligocene**				
*Eosphaeroma margarum*	?Salt Formation France	?Rupelian	**—**	Martini (1972)
*Eosphaeroma obtusum* (von Meyer, 1858)	Sieblos Beds, Sieblos Formation, Germany	Rupelian	**—**	von Meyer (1858); Martini (1969)
**Pliocene**				
*Jaliscosphaera pliocenica* García-Vázquez et al., 2023	“Paleolago Amatitán”, Mexico	Zanclean﻿–Piacenzian	**—**	García-Vázquez et al., (2023)

## Conclusion

We provide a morphological, taphonomic, and paleoecological assessment of *Archaeoniscus brodiei*—isopods from the Lower Cretaceous Durlston Formation. Examining two clusters preserved on the same slab, we identify novel anatomical data, highlighting that further examination of this species is needed. Geochemical examination of *A. brodiei* individuals using EDS illustrates that the isopods were preserved through phosphatisation under hypoxic conditions, and then likely experienced late-stage iron remobilization. The presence of two clusters evidences gregarious events— likely sheltering or mating—that were preserved during algal bloom-induced hypoxic shifts. This underscores the role of fluctuating anoxic conditions in preserving these rare palaeoecological events.

## Supplementary Information

Below is the link to the electronic supplementary material.

Fig. 7 Supplemental Figure 1: Examined slab showing two clusters of Archaeoniscus brodiei incolour. AMNH FI 15488.

High resolution image (EPS 52759 KB)
