# Supplementary figures and images for: Exploring the morphology and taphonomy of Archaeoniscus brodiei—a gregarious, Early Cretaceous isopod
Source: Naturwissenschaften. 2025 Feb 10;112(2):16. doi: 10.1007/s00114-025-01962-8 (PMC11811265; doi:10.1007/s00114-025-01962-8)

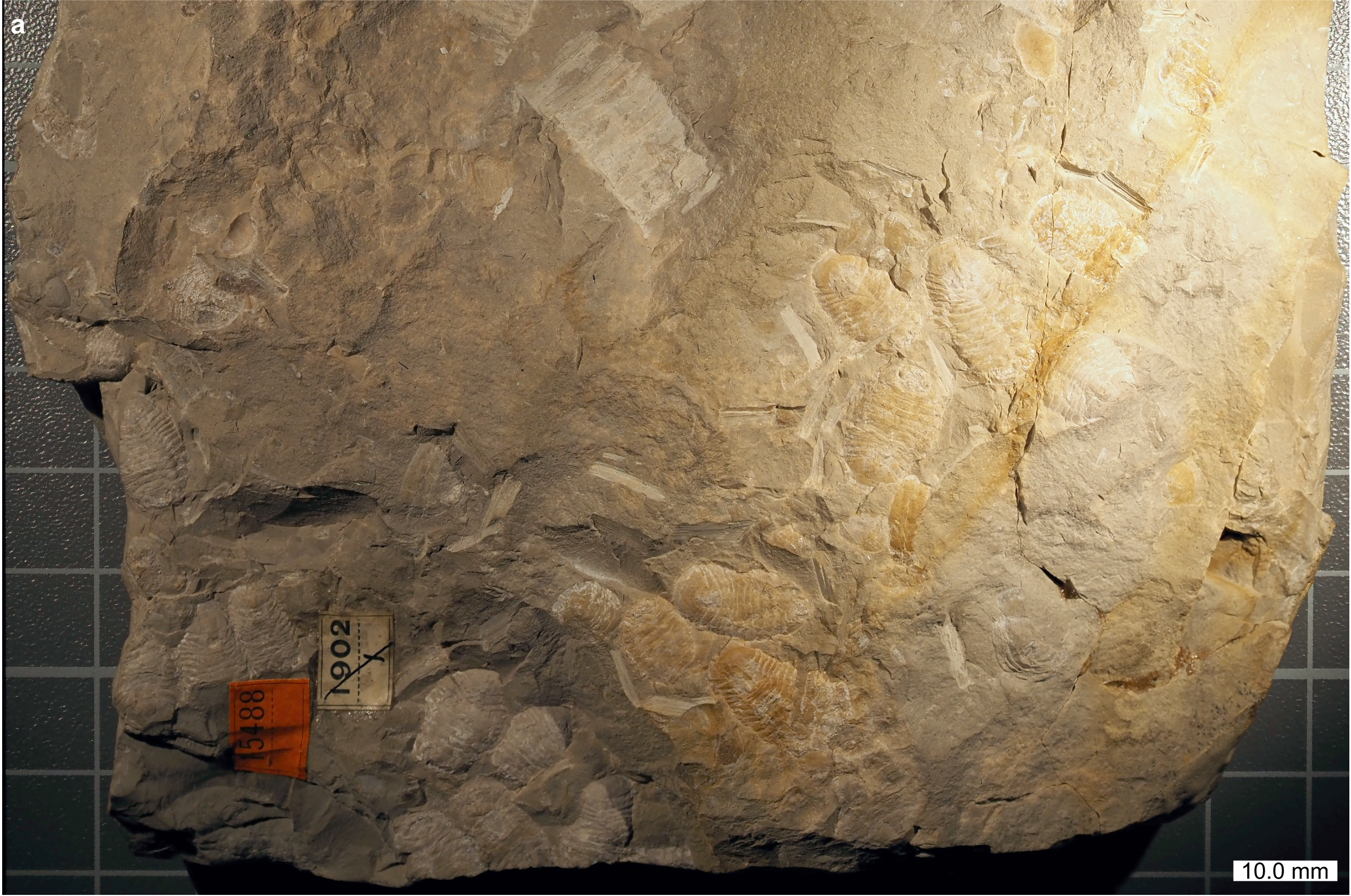

Supplement: Supplementary file 1 — Supplemental Figure 1: Examined slab showing two clusters of Archaeoniscus brodiei incolour. AMNH FI 15488. [file 114_2025_1962_Fig7_ESM.png]
